# Excitation, detection, and electrostatic manipulation of terahertz-frequency range plasmons in a two-dimensional electron system

**DOI:** 10.1038/srep15420

**Published:** 2015-10-21

**Authors:** Jingbo Wu, Alexander S. Mayorov, Christopher D. Wood, Divyang Mistry, Lianhe Li, Wilson Muchenje, Mark C. Rosamond, Li Chen, Edmund H. Linfield, A. Giles Davies, John E. Cunningham

**Affiliations:** 1 School of Electronic and Electrical Engineering, University of Leeds, Woodhouse Lane, Leeds LS2 9JT, United Kingdom

## Abstract

Terahertz frequency time-domain spectroscopy employing free-space radiation has frequently been used to probe the elementary excitations of low-dimensional systems. The diffraction limit, however, prevents its use for the in-plane study of individual laterally-defined nanostructures. Here, we demonstrate a planar terahertz frequency plasmonic circuit in which photoconductive material is monolithically integrated with a two-dimensional electron system. Plasmons with a broad spectral range (up to ~ 400 GHz) are excited by injecting picosecond-duration pulses, generated and detected by a photoconductive semiconductor, into a high mobility two-dimensional electron system. Using voltage modulation of a Schottky gate overlying the two-dimensional electron system, we form a tuneable plasmonic cavity, and observe electrostatic manipulation of the plasmon resonances. Our technique offers a direct route to access the picosecond dynamics of confined electron transport in a broad range of lateral nanostructures.

Picosecond time-resolved measurements of low-dimensional semiconductors can reveal a diverse range of physical phenomena. Typically, a device containing a two-dimensional electron system (2DES) is subjected to free-space propagating terahertz (THz) radiation (100 GHz < *f *< 5 THz), and either the transmitted THz response and/or the rectification response of the 2DES is then used to determine information about the system. Such experiments have provided information on, for example, coherent cyclotron resonance in a 2DES^1^,^2^, ultrastrong light-matter interactions between inter-Landau level transitions of a 2DES and the photonic modes of artificial resonators^3^,^4^, THz-wave modulation at room temperature^5^,^6^,^7^, and recently, the formation of plasmonic crystals using a gate-controlled 2DES^8^,^9^. The latter is particularly exciting, since the plasmonic cavity resonances in 2DESs on length scales of a few microns occur in the THz frequency range, offering the possibility of fabricating plasmonic circuits that can be used to manipulate THz signals.

Another class of experiments involves the planar integration of a 2DES into THz waveguides, which allows pulses to be either directly coupled into the system by ohmic contacts, using a flip-chip arrangement^10^, or coupled by proximity to a nearby THz waveguide, where they are exposed to and interact with the evanescent THz electric field^11^. In both these cases, electrical pulses are usually guided along a lithographically-defined, sub-wavelength transmission line structure formed on a separate substrate, before interacting with the 2DES. The in-plane nature of these techniques provides an enhanced interaction between the THz signal and the low-dimensional system relative to that achieved through free-space coupling. Such techniques have allowed ultrafast ballistic picosecond transport^10^ and magnetoplamon resonances^11^,^12^ to be studied.

Recently, we introduced an alternative technique in which growth-optimized LT-GaAs (providing THz-bandwidth pulse excitation and detection) and a high mobility 2DES channel are integrated in a single molecular beam epitaxy (MBE) wafer^13^. Here, we demonstrate that such integrated structures can be used to form broadband (up to ~400 GHz) on-chip plasmonic circuits capable of the in-plane excitation, detection, and electrostatic manipulation of 2D plasmons in quantum-confined 2DESs. The dynamic evolution of plasmon resonances in the gated 2DES region, controlled by an applied voltage, is recorded with a few-picosecond time resolution. Our methodology thus opens up a wide range of possible experiments in which broadband pulsed THz radiation is used to probe individual mesoscopic or nanoscale systems defined lithographically in a 2DES, rather than ensembles.

## Results

### Schematic and principle

**Figure 1:**
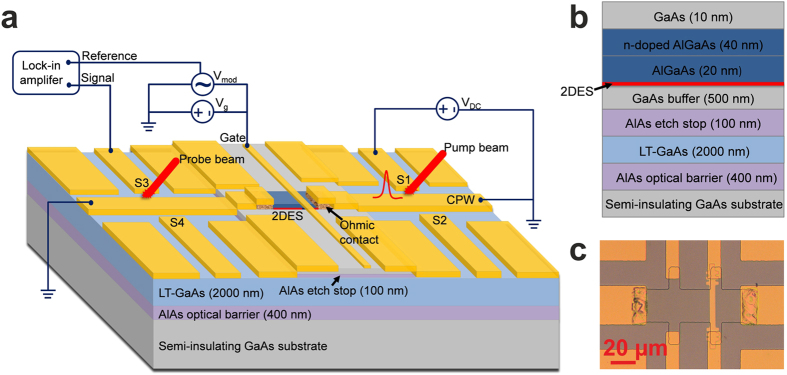
Diagram of the THz 2D plasmonic circuit. (**a**) Schematic diagram of the THz plasmonic circuit and the measurement arrangement for gate-modulation signals. S1/S2 and S3/S4 are two pairs of PC switches formed on opposite sides of the 2DES mesa, which are used to generate or detect the THz pulses; pulses are generated by application of a bias while under illumination by a 800 nm pulsed Ti:sapphire laser, while detection is achieved by measuring the generated photocurrent as a function of optical path time delay. (**b**) The layer structure of the wafer monolithically integrating the LT-GaAs and GaAs/AlGaAs heterostructure containing the 2DES (red region). (**c**) Microscopic image of the 2DES mesa. A 4.4-μm-long metallic gate was located on top of the 2DES mesa, and the widths of the ungated regions on either side of the gate were 19.7 μm and 48.9 μm.

A diagram of our THz 2D plasmonic circuit, in which the photoconductive material of LT-GaAs is monolithically integrated with 2DES, is shown in Fig. 1a. The device was fabricated from an MBE wafer (Fig. 1b), which comprised a layer of LT-GaAs along with a GaAs/AlGaAs heterostructure containing a 2DES (see further details in Methods). Two pairs of photoconductive (PC) switch contacts were then defined on the LT-GaAs layer, after it was subjected to a selective wet-etch to remove the 2DES and expose the underlying photoconductive LT-GaAs layer. A coplanar waveguide (CPW) guides THz pulses generated from, for example, PC switch S1, to an ohmic contact that is used to inject the picosecond pulses into the (73-μm-long) 2DES mesa. When the propagating THz pulse arrives at this ohmic contact, a portion of the pulse energy is injected into the 2DES, transmitted through the 2DES, and then exits through a second ohmic contact, before being coupled into the adjacent section of CPW. The first ohmic contact also reflects a portion of the propagating pulse. The time-resolved reflected or transmitted signals are then sampled at S2 or S3/S4, respectively. As shown in Fig. 1c, a 4.4-μm-wide metal gate was defined on the top of the 2DES mesa. A negative gate voltage (*V*_*g*_) applied to this gate was used to deplete carriers and so tune the electron concentration (*n*_*s*_) in the 2DES underneath. The voltage (*V*_*th*_) required to deplete carriers completely underneath the gate at 4 K after illumination was ~−3.0 V (see Supplementary Note 1 and Figure S1).

Our 2DES mesa supports the propagation of 2D plasmons at THz frequencies^14^,^15^. Therefore, when a THz pulse is injected into the 2DES, 2D plasmons are excited over a broad frequency range, and the 2DES acts as a plasmonic transmission line. The plasmonic dispersion relation in a 2DES is given by:




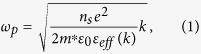




where *e* and m^*^ are the charge and effective mass of electrons in GaAs, respectively, *ε*_0_ is the vacuum dielectric permittivity, *ε*_*eff  *_(*k*) is the effective relative permittivity, and *k* is the plasmon wave number^16^,^17^,^18^. Unlike bulk plasmons, the resonance frequency of 2D plasmons depend on geometric size. In order to excite a resonant plasmonic mode, *k* must satisfy the condition *k *= *nπ/L*, where *n *= 1, 2, 3…, and *L* is the length of the plasmonic cavity. The phase velocity of plasmons is then obtained using *v*_*p *_= *ω*_*p*_/*k*. In the ungated regions, the 2DES behaves as a dispersive, single-wire-like plasmonic transmission line^19^. In the gated region, however, the metallic gate screens the Coulombic restoring force, which has the effect of reducing the acceleration of electrons driven by the exciting THz electric field. The plasmons in this region therefore have a much lower *v*_*p*_ in comparison with the ungated areas. The effective permittivity *ε*_*eff  *_(*k*) in the gated region is given by *ε*_*eff*_  (*k*) = [*ε*_2 _+ *ε*_1_coth(*kd*)]/2, where *ε*_1_ and *ε*_2_ are the relative permittivity of AlGaAs and GaAs, respectively, and *d* is the distance from the metallic gate to the 2DES^18^. The plasmon dispersion can hence be written:




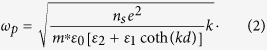




If the screening effect is strong (when *kd*→0), the transmission line effectively then acts as a parallel-plate, plasmonic waveguide, supporting a dispersionless transverse electromagnetic (TEM) mode^8^,^17^.

### THz pulse propagation in the 2DES

**Figure 2:**
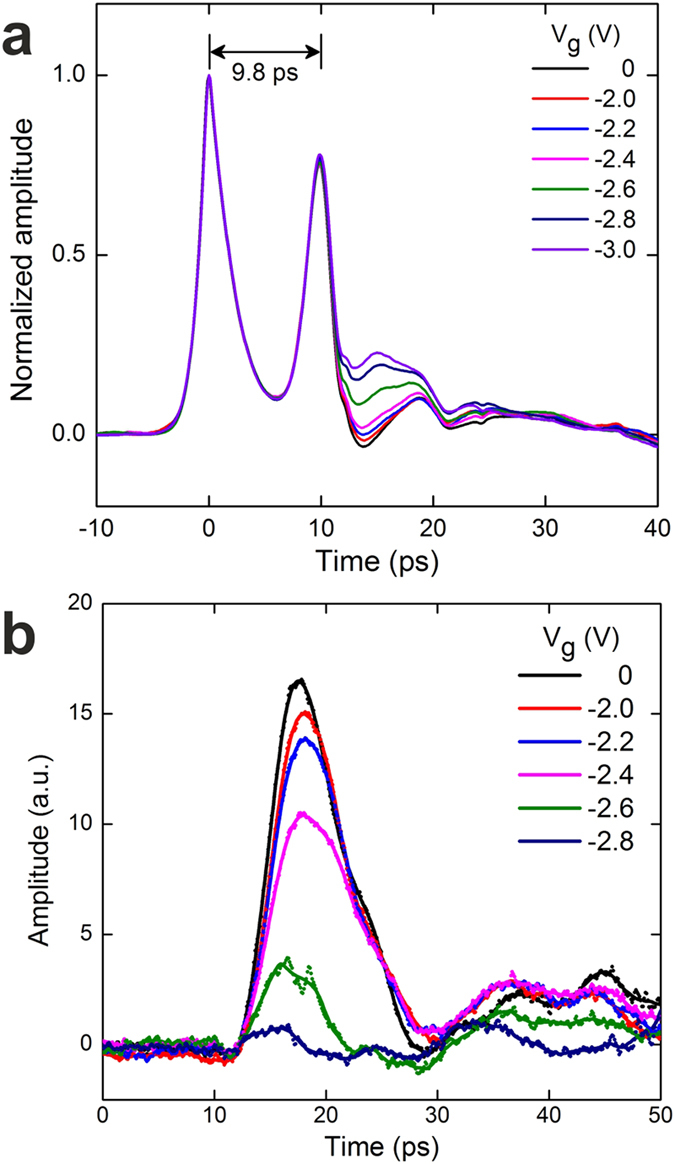
Input and transmitted THz pulse through a 2DES channel. (**a**) The measured input (and reflected) pulses for different *V*_*g*_ at 4 K. The THz pulse is generated by S1 and detected by S2. (**b**) The measured transmitted signals through the 2DES for different *V*_*g*_ at 4 K. The coplanar mode THz pulse is generated by biasing both S3 and S4, with detection by S2.

To investigate the injection into and propagation of THz pulses through the 2DES, we measured the input and transmitted pulses using what is, in effect, an on-chip THz time-domain spectrometer^20^,^21^. Initial measurements were performed in a continuous-flow helium cryostat at 4 K (see Supplementary Note 2 and Figure S2). Figure 2a shows the measured input and reflected pulses as a function of *V*_*g*_. The first peak observed at 0 ps is the input pulse generated by S1, and detected by S2 through the conductive coupling of the two adjacent PC switches across the centre conductor. The second peak, centred at 9.8 ps, is the signal reflected from the ohmic contact closest to S1/S2. When a THz pulse arrives at this ohmic contact, it is partly reflected from the CPW/2DES interface, whilst a portion of the signal is injected into the 2DES mesa. As *V*_*g*_ is reduced from 0 V to *V*_*th*_ (-3.0 V), a ‘shoulder’ appears on the reflected time-domain signal. The amplitude of this feature increases with decreasing *V*_*g*_, and saturates at *V*_*th*_. We attribute the ‘shoulder’ feature to the increased reflection of the signal propagating in the 2DES by the elevated barrier under the gated region when a negative voltage is applied. When *V*_*g *_= 0 V, and since *v*_*p*_ in gated region is lower than that in ungated region, the mismatch of *k (k *= *ω/v*_*p*_) leads to the formation of a barrier. As *V*_*g*_ decreases, *n*_*s*_ and the corresponding *v*_*p*_ in the gated region also both decrease. As a result, the mismatch of *k* between the gated and ungated regions increases, which results in the observed increase in reflection from the interface between gated and ungated regions. Once *V*_*g*_ reaches *V*_*th*_, the 2DES channel is fully pinched off, so the amplitude of the reflection cannot increase any further.

The CPW allows two dominant quasi-TEM modes of propagation: a coplanar and a slotline mode. For the input pulse measurement, the signal launched from a single PC switch is a mixture of two modes. In our structure, a pair of PC switches (S3 and S4) is located at either side of the CPW centre conductor, allowing mode-selective excitation of the waveguide by the choice of PC switch biases^21^ (see Supplementary Note 3). We found that, by decreasing *V*_*g*_ from 0 V to *V*_*th*_, the transmitted coplanar mode signal showed a strong dependence on *V*_*g*_, whereas the transmitted slotline mode component showed no such dependence (Supplementary Figures S3b,c), indicating that only the coplanar mode is efficiently injected into the 2DES. By decreasing *V*_*g*_ from 0 V to *V*_*th*_, the transmitted coplanar mode signal showed a strong dependence on *V*_*g*_. When the 2DES channel is pinched-off (*i.e.* for *V*_*g *_≤ -3 V), there is still a sizable signal, caused by a crosstalk signal resulting from the capacitive coupling of pulses between the ohmic contacts and the coupling between two ungated plasmon cavities^22^. This parasitic signal does not depend on *V*_*g*_, however, allowing us to obtain the pulse transmitted through the 2DES channel by subtraction of the crosstalk signal for a pinched-off channel (at *V*_*g *_= -3 V, T = 4 K). As shown in Fig. 2b, the obtained transmitted signal does not alter significantly between 0 V and -2 V. However, as *V*_*g*_ approaches *V*_*th*_, the mismatch in *k* between the gated and ungated regions greatly increases, owing to the decrease of *n*_*s*_ and *v*_*p*_ in the gated region (Supplementary Figure S4), leading to a sharp decrease in transmission. In addition, the full-width-half-maximum (FWHM) of the transmitted pulse at *V*_*g *_= 0 V is approximately 9 ps, which is much larger than that of the input pulse width (~1.5 ps). The contribution to this pulse broadening within the CPW region is 1.2 ps, judged by comparing the FWHM of reflected pulse (~2.7 ps) which propagates the same distance within CPW as the transmitted pulse (Fig. 2a), with this input pulse width. Therefore, the observed pulse broadening (of ~9–1.5 ps = 7.5 ps) is mainly caused by the strong dispersion of plasmons in the 2DES, and especially in the ungated regions where their dispersion is greater^18^,^23^.

### Electrostatic modulation of 2D plasmons

The dynamics of 2D plasmons in the 2DES can be obtained by measurements of the transmitted pulse as a function of *V*_*g*_. However, comparative data obtained by subtracting pulses measured at different times exhibited poor signal-to-noise ratio (SNR), owing to a very slow drift of the laser power and/or focus position. We therefore employed a different technique for more detailed measurements, whereby a small AC signal (*V*_*mod*_) was superimposed onto the DC gate bias, allowing lock-in detection (see Fig. 1a). The time-resolved current of the transmitted pulse is denoted by *I*(*t*,*V*_*g*_), and its change (Δ*I*(*t*,*V*_*g*_)) with the swing of gate bias around the average voltage *V*_*g*_, *i.e. ΔI(t*,*V*_*g*_)/*ΔV*_*g*_, is extracted using standard lock-in techniques. If the amplitude of *V*_*mod*_ is sufficiently small, the measured signal is equivalent to *dI(t*,*V*_*g*_)/*dV*_*g*_ (see Supplementary Note 5). By altering *V*_*g*_ at a fixed *V*_*mod*_, changes in the transmitted THz signal as a function of *V*_*g*_ could then be recorded. This gate-modulation technique provided an improvement in SNR of more than 50 times (see Supplementary Note 6 and Figure S6). In order to interpret our gate-modulation results, a full analytical model was developed (see Supplementary Note 7). Based on this model, we find that a resonant excitation signal is selected by the gate-modulation, allowing the resonance frequency of plasmons in the cavity to be extracted.

**Figure 3:**
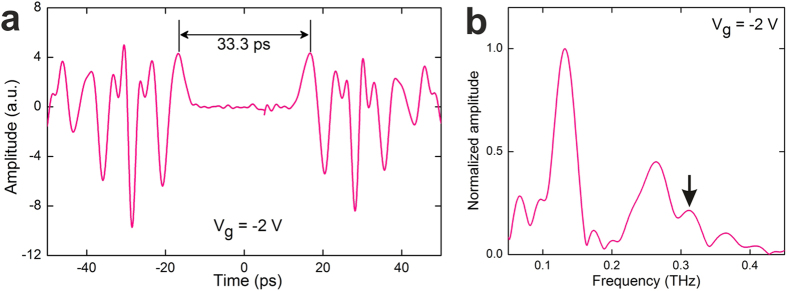
Modulation of 2D plasmons in a 2DES channel. (**a**) A superposition of the measured gate-modulation signals as a function of time for *V*_*g *_= -2.0 V, in which the THz pulses propagate from S1 to S3 (positive time domain) and from S3 to S1 (negative time domain). (**b**) The FFT spectrum of the gate-modulation signal measured at *V*_*g *_= -2.0 V. The resonance at 311 ± 6 GHz is indicated by a black arrow.

The detailed gate-modulation measurements were performed in a closed-cycle He_3_/He_4_ dilution refrigerator (see Methods for details)^13^. Figure 3a shows the gate-modulation signals obtained at *V*_*g *_= -2 V, when a PC switch bias (*V*_*DC*_) of +5 V was applied to S1 to generate a picosecond pulsed signal propagating towards S3. In order to remove high-frequency noise in the data, without losing useful information, the measured signals were passed through a digital low-pass filter with a cut-off frequency of 0.6 THz, chosen to be higher than the upper frequency of the gate-modulated signals (~0.4 THz). The time taken for the pulse to propagate through the structure corresponds to the sum of the transit time in the CPW (*t*_*CPW*_), and that in the 2DES (*t*_2*DES*_). Measurement of pulses generated at S1 and measured at S3 were superposed with a measurement of the reverse-transmitted THz signal (*i.e.* propagating from S3, biased at +5 V, to S1) revealing a total round-trip time, Δ*t *= 2(*t*_*CPW *_+ *t*_*2DES*_), of 33.3 ps (Fig. 3a). The measured gate-modulation signals were found to be symmetric for the two propagation directions (which was verified by swapping the excitation and detection switches). The *t*_*CPW*_ was found to be ~9.8 ps, by measuring the time delay between the input pulse and its reflection from the CPW/2DES interface (Fig. 2a). Thus, the *t*_2*DES*_ is ~6.9 ps. The corresponding average pulse velocity in the 2DES is ~1.1 × 10^7^ m/s, which is an order of magnitude smaller than that in the CPW (~1.1 × 10^8^ m/s) and close to the expected plasmon velocity in the 2DES, supporting our assumption of plasmonic excitation. In addition, the transmitted signals shown in Fig. 3a are composed of clear periodic oscillations. When a picosecond pulse is injected into the 2DES, the frequency components of the pulse that satisfy the Fabry-Perot (FP) resonance conditions of the gated or ungated plasmonic cavities are trapped, and undergo leaky oscillations within the cavity. Since *n*_*s*_ in the gated cavity is tuned by *V*_*g*_, the resonant signals from the gated plasmonic cavity are strongly modulated by the DC component of *V*_*g*_. Using lock-in technique, the modulated resonant excitation signal is recorded, and the corresponding resonance frequency and decay of the excitation can be obtained, using our analytical model. In the corresponding fast Fourier transform (FFT) spectrum (Fig. 3b), the two strongest plasmon resonance frequencies observed are 132 ± 2 GHz and 264 ± 2 GHz, corresponding to the fundamental and second plasmonic mode of the gated plasmonic cavity, respectively. Using *n*_*s *_= 4.9 × 10^15^ m^−2^ at the same *V*_*g *_= -2 V, obtained from the *in situ* two-terminal magnetotransport measurement (see Supplementary Note 4), the predicted plasmon resonance frequencies in the gated region calculated using Equation (2) are 136 GHz (*n *= 1 and *k *= π/4.4 μm^−1^) and 264 GHz (*n *= 2 and *k *= 2π/4.4 μm^−1^), which are close to our measured values. The gated region of 2DES works as a FP cavity, and the gate length is equivalent to a half wavelength and wavelength for the fundamental and second modes, respectively. There is also a resonance mode around 311 GHz in Fig. 3b (indicated by black arrow), which can be attributed to the fundamental plasmon mode in the 19.7-μm-long ungated region (the calculated frequency using Eq. (1) is 306 GHz). That the ungated plasmon resonance signals can be extracted from these measurements is at first somewhat surprising, but this can be explained from our analytical model (see Supplementary Note 7). Unlike the modes formed by the gated cavity, this resonance does not change frequency when *V*_*g*_ is altered. However, as shown in Fig. 2b, the transmission of the broadband injected signal through the 2DES is also strongly modulated by the swing of *V*_*g*_ when *V*_*g*_ is below -2 V. Therefore, the amplitude of oscillation in the ungated cavity is also modulated, allowing this resonance mode to also be detected using our gate-modulation method. Aside from these three main resonance modes, there are several other weaker resonance peaks in the whole spectrum, which can be classified as coupled modes of two neighbouring cavities for low frequency (<100 GHz) resonances, and higher order modes.

**Figure 4:**
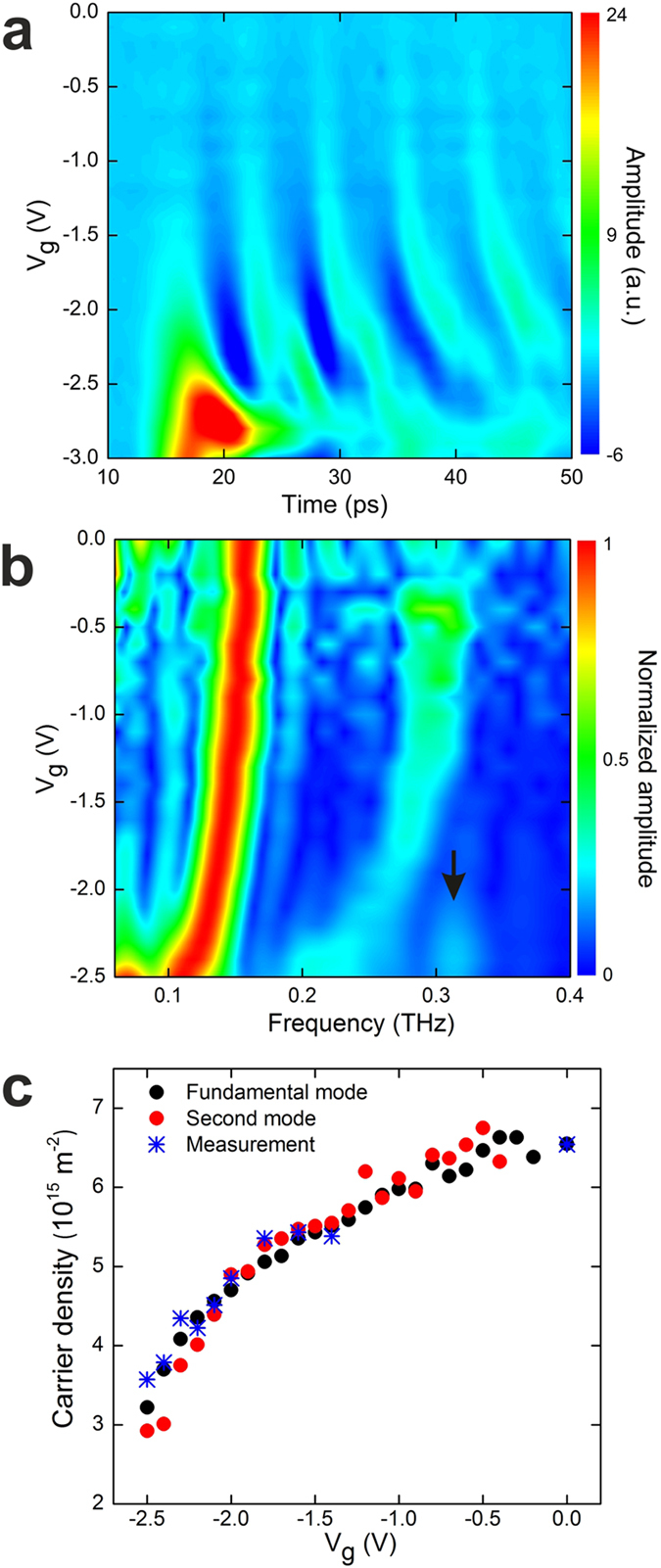
Electrostatic manipulation of 2D plasmon resonances. (**a**) Colour-scale plot of the measured gate-modulation signals plotted as a function of time and *V*_*g*_. (**b**) Colour-scale plot of the normalized FFT spectra for gate-modulation signals measured as a function of frequency and *V*_*g*_. The resonance around 311 GHz for *V*_*g *_≤ -2.0 V is indicated by a black arrow. (**c**) The *n*_*s*_ in the gated region as a function of *V*_*g*_. These are calculated using the measured frequencies of fundamental (black dots) and second (red dots) plasmon modes and from the two-terminal magnetotransport measurements of the same 2DES mesa (blue asterisks).

To investigate the dependence of the plasmon resonances on *V*_*g*_, we measured the time-domain profiles of the gate-modulation signals as a function of *V*_*g*_ (Fig. 4a). As *V*_*g*_ is swept toward *V*_*th*_, *n*_*s*_ in the gated region decreases, and correspondingly the plasmon resonance frequency in the gated region is reduced. This results in an increase in the observed plasmonic oscillation period of the time domain signals. In the corresponding FFT spectra (Fig. 4b), the frequency of the first gated plasmon mode is tuned from 159 GHz to 111 GHz by sweeping *V*_*g*_ from -0.4 V to -2.5 V, and the red-shift is 48 GHz. Meanwhile, the frequency of the second mode experiences a red-shift of 97 GHz from 302 GHz to 205 GHz. The values of resonance frequencies and red-shift for the second mode are approximately twice that of the first mode. From the measured frequencies of the gated plasmon modes in Fig. 4b, we calculated *n*_*s*_ for each value of *V*_*g*_ using Equation (2) and compared them with the measured *n*_*s*_ from magnetotransport measurements, as shown in Fig. 4c, and found good agreement between these values.

## Conclusion

We have demonstrated an on-chip THz system that allows the picosecond-duration excitation, detection and electrostatic manipulation of 2D plasmons. Using a gate-modulation technique, we observed the picosecond-resolved time evolution of confined 2D plasmons. This work not only provides a useful technique with which to study the ultrafast THz response and carrier dynamics of low-dimensional semiconductor structures, but also has potential future application in the development of more complex plasmonic circuits capable of manipulating THz waves. We note that in the system studied here, the properties of the 2DES could in principle be explored at even higher frequencies if the bandwidth of the plasmonic circuit could be expanded, with substrate thinning of the integrated wafer being one potential route to achieve this goal^24^.

## Methods

### Device fabrication and characterization

The wafer from which the device was fabricated was grown by MBE with the layer structure shown in Fig. 1b. To obtain *n*_*s*_ and *μ* of the 2DES, a Hall bar was fabricated, and magnetotransport measurements were undertaken in a 1.2 K helium bath cryostat with a superconducting magnet. The *n*_*s*_ and *μ* of the 2DES in the dark were 3.66 × 10^15^ m^−2^ and 48.7 m^2^/(V·s), respectively. After illumination by a HeNe laser, *n*_*s *_= 6.26 × 10^15^ m^−2^ and *μ *= 89.5 m^2^/(V·s) were obtained. The corresponding momentum relaxation time (*τ*_*p*_) was calculated to be 33 ps.

To fabricate the device, first a 100 × 25 μm^2^ mesa containing the 2DES was defined using a sulphuric acid etch (H_2_SO_4_:H_2_O_2_:H_2_O = 1:8:70) to a depth of 100 nm. Next, two 12.5 × 30 μm^2^ ohmic contacts to the 2DES were formed by depositing Au-Ge-Ni alloy (200 nm) at either end of the 2DES region, followed by annealing at 430 °C for 80 seconds. A continuous 4.4-μm-wide gate was then defined on top of the mesa using electron-beam lithography, and subsequent deposition of Ti/Au (10/60 nm)) by electron-beam evaporation. To expose the underlying PC LT-GaAs layer, the region immediately surrounding the 2DES was protected using photoresist (Shipley S1813), and the exposed surface etched in a selective etchant mixed from 50% citric acid and 30% H_2_O_2_ in a 3:1 ratio, down to an AlAs etch-stop layer, which was subsequently removed in dilute HF (5% concentration) to reveal a smooth LT-GaAs surface. A CPW structure, designed to incorporate two pairs of symmetric PC switches, was next defined by photolithography, and metallised via electron-beam evaporation of Ti/Au (10/150 nm). In the CPW structure, the widths of the centre conductor and gaps were 30 μm and 20 μm, respectively. The CPW centre conductor was aligned to the ohmic contacts formed previously on the 2DES. Corresponding breaks in the ground plane were incorporated, both to suppress crosstalk, and to allow contact to be made to the 2DES gate.

### Cryogenic measurement in 4K cryostat and dilution refrigerator

For measurements performed at 4 K, the sample was mounted in a continuous-flow liquid helium cryostat, with optical access provided through a z-cut quartz window^20^. The laser beams were focused through the quartz window onto the PC switches. The average laser power of both the pump and probe beams was fixed at 10 mW.

For measurements below 4 K, and in magnetic fields, the sample was mounted on a holder attached to mixing plate of a closed-cycle He^3^/He^4^ dilution refrigerator, located at the centre of a superconducting magnet. The experimental setup comprised *in-situ* piezoelectric stages used to position the laser beams transmitted via fibre optics on the sample surface, as discussed in detail in ref. 13. Both the pump and probe laser beam average power were set to be 2 mW, which resulted in heating of the sample stage to around 2 K, measured using an embedded RuO_2_ thermometer immediately adjacent to the device.

### Experimental setup for input and transmitted pulse measurements

For input pulse measurements (see Supplementary Figure S2a), shunt interconnects were used to apply a DC bias to a PC switch (S1) which, when illuminated using a 100 fs pulsed, 800 nm near infrared (NIR) laser, generated a THz pulse that was coupled into the overlaid CPW structure. The unbiased PC switch immediately adjacent to S1 (S2) was illuminated by a time-delayed and mechanically chopped NIR laser beam, allowing time-resolved detection of the THz pulse launched into the CPW, and of reflected pulses generated from the 2DES. The current flowing in S2 was then extracted using standard lock-in techniques, with an optical chopper used to provide reference.

For transmitted pulse measurements (see Supplementary Figure S3a), a pair of PC switches (S3 and S4) were both biased positively with respect to the centre conductor and illuminated by a defocused pump laser beam, in order to generate a coplanar mode within the waveguide^21^. The PC switch on the opposite side of the 2DES mesa (S1), illuminated by the chopped probe beam, was then used to detect pulses which had propagated along the CPW and through the attached 2DES. In order to extract measurements of pulses transmitted through the 2DES mesa, the crosstalk signal between two ends of CPW was first measured by pinching off the 2DES channel (*V*_*g *_= -3 V). The signals transmitted at different *V*_*g*_ were then measured. By subtracting the crosstalk signal from each measured signal, the pulses transmitted through the 2DES were obtained.

### Experimental setup for gate-modulation measurement

As shown in Fig. 1a, S1 and S3 were illuminated by the pump and probe laser beams, respectively. The THz pulse was generated by S1, which was biased by a DC voltage. Both *V*_*g*_ and *V*_*mod*_ were applied on the metallic gate simultaneously to control *n*_*s*_ underneath the gated region. *V*_*g*_ and *V*_*mod*_ (the latter an 87 Hz sinusoidal wave, with 25 ~ 100 mV rms amplitude) were supplied by a digital-to-analogue converter and an ultra-pure sinewave oscillator, respectively. The gate-modulation signal was measured at S3 using a lock-in amplifier, whose reference signal was provided via the synchronous output of the sinewave oscillator. To facilitate the alignment of the laser beam in the dilution refrigerator, we use one rather than two PC switches to generate the THz pulse. Though the pulse generated from a single PC switch is a mixture of coplanar and slotline modes, only the coplanar mode signal could pass through the 2DES and be modulated by *V*_*mod*_ (see Supplementary Note 3). In this configuration, the THz pulse propagates through the 2DES in the direction from S1 to S3. To measure the gate-modulation signal for a THz pulse propagating in the opposite direction, the electrical connections of S1 and S3 were swapped whilst maintaining fixed laser beam positions, and an equivalent measurement was taken. In this case, the laser beams swap functionality (*i.e.* the pump beam is now the probe beam, and *vice versa*).

## Additional Information

**How to cite this article**: Wu, J. *et al.* Excitation, detection, and electrostatic manipulation of terahertz-frequency range plasmons in a two-dimensional electron system. *Sci. Rep.* **5**, 15420; doi: 10.1038/srep15420 (2015).

## Supplementary Material

Supplementary Information

## References

[b1] Wang X. *et al.* Terahertz time-domain magnetospectroscopy of a high-mobility two-dimensional electron gas. Opt. Lett. 32, 1845–1847 (2007). 17603589 10.1364/ol.32.001845

[b2] Huggard P. G. *et al.* Coherent control of cyclotron emission from a semiconductor using sub-picosecond electric field transients. Appl. Phys. Lett. 71, 2647–2649 (1997).

[b3] Scalari G. *et al.* Ultrastrong coupling of the cyclotron transition of a 2D electron gas to a THz metamaterial. Science 335, 1323–1326 (2012). 22422976 10.1126/science.1216022

[b4] Maissen C. *et al.* Ultrastrong coupling in the near field of complementary split-ring resonators. Phys. Rev. B 90, 205309 (2014).

[b5] Kleine-Ostmann T., Dawson P., Pierz K., Hein G. & Koch M. Room-temperature operation of an electrically driven terahertz modulator. Appl. Phys. Lett. 84, 3555–3557 (2004).

[b6] Sensale-Rodriguez B. *et al.* Broadband graphene terahertz modulators enabled by intraband transitions. Nat. Commun. 3, 780 (2012). 22510685 10.1038/ncomms1787

[b7] Lee S. H. *et al.* Switching terahertz waves with gate-controlled active graphene metamaterials. Nat. Mater. 11, 936–941 (2012). 23023552 10.1038/nmat3433

[b8] Dyer G. C. *et al.* Inducing an incipient terahertz finite plasmonic crystal in coupled two dimensional plasmonic cavities. Phys. Rev. Lett. 109, 126803 (2012). 23005973 10.1103/PhysRevLett.109.126803

[b9] Dyer G. C. *et al.* Induced transparency by coupling of Tamm and defect states in tunable terahertz plasmonic crystals. Nat. Photon. 7, 925–930 (2013).

[b10] Shaner E. A. & Lyon S. A. Picosecond time-resolved two-dimensional ballistic electron transport. Phys. Rev. Lett. 93, 037402 (2004). 15323867 10.1103/PhysRevLett.93.037402

[b11] Shaner E. A. & Lyon S. A. Time-resolved impulse response of the magnetoplasmon resonance in a two-dimensional electron gas. Phys. Rev. B 66, 041402(R) (2002).

[b12] Shaner E. A., Lyon S. A. & Engel L. W. Picosecond electrical excitation of a two-dimensional electron gas. Proc. SPIE 5352, Ultrafast Phenomena in Semiconductors and Nanostructure Materials VIII. San Jose, CA, USA, 10.1117/12.533181. 364–371 (2004 June 16)

[b13] Wood C. D. *et al.* On-chip terahertz spectroscopic techniques for measuring mesoscopic quantum systems. Rev. Sci. Instrum. 84, 085101 (2013). 24007101 10.1063/1.4816736

[b14] Burke P. J., Spielman I. B., Eisenstein J. P., Pfeiffer L. N. & West K. W. High frequency conductivity of the high-mobility two-dimensional electron gas. Appl. Phys. Lett. 76, 745−747 (2000)

[b15] Andress W. F. *et al.* Ultra-subwavelength two-dimensional plasmonic circuits. Nano Lett. 12, 2272−2277 (2012). 22494364 10.1021/nl300046g

[b16] Stern F. Polarizability of a two-dimensional electron gas. Phys. Rev. Lett. 18, 546–548 (1967).

[b17] Allen S. J. Jr., Tsui D. C. & Logan R. A. Observation of the two-dimensional plasmon in silicon inversion layers. Phys. Rev. Lett. 38, 980–983 (1977).

[b18] Shur M. Plasma wave terahertz electronics. Electron. Lett. 46, s18–s21 (2010).

[b19] Aizin G. R. & Dyer G. C. Transmission line theory of collective plasma excitations in periodic two-dimensional electron systems: Finite plasmonic crystals and Tamm states. Phys. Rev. B 86, 235316 (2012).

[b20] Wood C. *et al.* On-chip photoconductive excitation and detection of pulsed terahertz radiation at cryogenic temperatures. Appl. Phys. Lett. 88, 142103 (2006).

[b21] Zamdmer N., Hu Q., Verghese S. & Förster. A. Mode-discriminating photoconductor and coplanar waveguide circuit for picosecond sampling. Appl. Phys. Lett. 74, 1039 (1999).

[b22] Ernst G., Haug R. J., Kuhl J., von Klitzing K. & Eberl K. Acoustic edge modes of the degenerate two-dimensional electron gas studied by time-resolved magnetotransport measurements. Phys. Rev. Lett. 77, 4245 (1996). 10062485 10.1103/PhysRevLett.77.4245

[b23] Yoon H., Yeung K. Y., Kim P. & Ham D. Plasmonics with two-dimensional conductors. Philos. Trans. A Math. Phys. Eng. Sci. 372, 20130104 (2014). 24567472 10.1098/rsta.2013.0104 PMC3928902

[b24] Russell C. *et al.* Spectroscopy of polycrystalline materials using thinned-substrate planar Goubau line at cryogenic temperatures. Lab on a Chip 13, 4065–4070 (2013). 23963477 10.1039/c3lc50485a

