# Supplementary material for: Excitation, detection, and electrostatic manipulation of terahertz-frequency range plasmons in a two-dimensional electron system
Source: Sci Rep. 2015 Oct 21;5:15420. doi: 10.1038/srep15420 (PMC4614073; doi:10.1038/srep15420)
Supplement: Supplementary Information [file srep15420-s1.pdf]

## Supplementary Information

# Excitation, detection, and electrostatic manipulation of terahertz-frequency range plasmons in a two-dimensional electron system

Jingbo Wu, Alexander S. Mayorov, Christopher D. Wood, Divyang Mistry, Lianhe Li, Wilson Muchenje, Mark C. Rosamond, Li Chen, Edmund H. Linfield, A. Giles Davies, and John E. Cunningham

School of Electronic and Electrical Engineering, University of Leeds, Woodhouse Lane, Leeds LS2 9JT, United Kingdom.

[*] j.e.cunningham@leeds.ac.uk

**Supplementary Note 1: Two-terminal conductance measurements of 2DES.**

When the sample was cooled to 2 K, we found that switching on the laser resulted in an increase in the 2DES conductance through scattering of light onto the sample, and that this increase remained even after the laser was switched off, owing to persistent conductivity effects [1]. To measure the 2DES two-terminal conductance after illumination, an AC voltage (100 μV rms, 83 Hz) was applied across the 2DES and centre conductor of the CPW, with a DC bias ($V_g$) applied to the metallic gate, and lock-in detection was used to measure the conductance. The combined DC conductance of the CPW centre conductor and 2DES under illumination at 2 K is plotted in Supplementary Figure S1. The pinch-off voltage of the 2DES ($V_{th}$), *i.e.* the voltage for which the carriers beneath the gated region are completely depleted, was found to be –3.0 V.

**Supplementary Note 2: $V_g$ dependence of the input and reflected THz pulses.**

The experimental arrangement used for the input and reflected pulse measurements is shown in Supplementary Figure S2a. The sample was mounted in a continuous-flow liquid helium cryostat, with the THz pulse generated at switch S1 and detected at S2. The average pump and probe beam powers were 10 mW, and $V_{DC}$ was set to be +20 V

The input and reflected pulses as a function of $V_g$ at 4 K were measured and each measured signal was normalized by the value of its first peak (input pulse) at 0 ps (Figure 2a of the main article). When $V_g$ is –3 V, the 2DES channel is pinched off, and the injected signal into the 2DES is completely reflected from the gated region. After subtracting the signal for $V_g$ = –3 V from each signal for different $V_g$, we extract the relative change of reflected signals from the gated region. As shown in Supplementary Figure S2b, there is almost no signal at the peak position of input pulse (0 ps) and reflected pulse from ohmic contact (9.8 ps), indicating that these signals do not depend on $V_g$. Conversely, a strong peak appears at the 'shoulder' in Figure 2a of the article, which increases monotonically in amplitude as $V_g$ decreases. As discussed in the main article, the increased reflection of the broadband THz signal with decreasing $V_g$ is due to the increased mismatch of wave number between gated and ungated region.

In Figure 2a of the main article, the delay time between the first and second peaks is 9.8 ps, which corresponds to the round-trip time of the THz pulse along the length of the CPW connecting S1 and S2 to the 2DES mesa. The length of this CPW is 550 μm, from which the THz pulse velocity is

calculated to be ~1.1×10$^8$ m/s in the CPW. Considering that the CPWs on the two sides of 2DES mesa are symmetric, the single-trip transit time of a pulse propagating in the CPW between S1/S2 and S3/S4 without the 2DES mesa is 9.8 ps.

**Supplementary Note 3: Transmitted coplanar mode and slotline mode signals.**

In the CPW, two main modes are supported: the coplanar mode and the slotline mode. There is significant difference in the field distribution between these two modes: for the coplanar mode, the electric field distribution is symmetric with respect to the CPW centre conductor, and the electric field lines lie between the centre conductor and the two adjacent ground planes, whilst for the slotline mode propagation, the electric field lines lie between the two ground planes, bypassing the centre conductor. The radiative losses of the slotline mode are much greater than of the coplanar mode [2,3].

A diagram of selective mode generation and transmitted pulse detection is shown in Supplementary Figure S3a. A defocused pump beam is used to illuminate the two PC switches on the same side of the 2DES (*e.g*. S3 and S4). When the two switches are biased with the same positive voltage ($V_{DC1}$ = $V_{DC2}$), a pure coplanar mode is generated and propagates in the CPW. For slotline mode generation, the polarity of one PC switch is altered, such that $V_{DC1} = -V_{DC2}$. The PC switch on the opposite side of the 2DES mesa and on either side of the CPW (*e.g*. S2 in Supplementary Figure S3a), which is illuminated by chopped probe beam, is used to detect the transmitted pulse in both cases. Under coplanar mode excitation, the transmitted pulses measured on both sides of the CPW (S1/S2) have the same pulse shape and polarity because of the symmetric field pattern of the coplanar mode. When the slotline mode is excited, the pulse shape measured on either side of CPW is nearly the same, but the polarity is opposite, indicating that the field pattern of slotline mode is asymmetric.

The transmitted coplanar mode and slotline mode signals for different $V_g$ at 4 K are shown in Supplementary Figure S3b,c. The measurements were taken in a continuous-flow liquid helium cryostat. The average pump and probe beam powers were maintained at 10 mW. The biases on PC switches of S3 and S4 were: $V_{DC1} = V_{DC2} = +20$ V for coplanar mode excitation, and $V_{DC1} = -V_{DC2} = +20$ V for slotline mode excitation. The transmitted coplanar mode signal exhibits an obvious dependence on $V_g$, with decreasing amplitude as $V_g$ is varied from 0 V to –3 V (Supplementary Figure S3b). This indicates that the THz pulse is interacting strongly with the 2DES, and the coplanar

mode transmission can therefore be controlled electrostatically by altering $V_g$. When the channel is pinched off ($V_g$ = –3 V), a strong, broad transmitted pulse is still observed, which is attributed to crosstalk between the two ends of the 2DES mesa. By subtracting the transmitted pulses obtained for different $V_g$ from the crosstalk signal measured at $V_g$ = –3 V, the transmitted pulses through 2DES mesa were obtained as shown in Figure 2b of the main article. In contrast, the transmitted slotline mode signals is almost unaffected as $V_g$ decreases from 0 V to –3 V as shown in Supplementary Figure S3c, indicating that the slotline mode THz pulse does not interact strongly with the 2DES. For the slotline mode propagating in the CPW, the electric field close to the centre conductor is much weaker than that of coplanar mode, and therefore the conversion efficiency from the slotline mode to 2D plasmons in the 2DES is low.

**Supplementary Note 4: Two-terminal magnetoresistance measurements and carrier density in the 2DES.**

For two-terminal magnetotransport measurements, a magnetic field was applied normal to the 2DES plane and swept from 0 to 2.5 T. Supplementary Figure S4a shows the two-terminal resistance of the 2DES measured as a function of magnetic field at $V_g$ = –2 V. The resistance of the leads and centre conductor of CPW were not subtracted. The resistance increases with magnetic field, and quantum Hall plateaux appear at high magnetic fields. For two-terminal measurements of a 2DES, the resistance in magnetic field depends on both diagonal and off-diagonal components of the resistivity tensor, and on the sample geometry. Therefore, the Shubnikov-de Hass (SdH) oscillations are not clearly resolved in Supplementary Figure S4a. By calculating the second derivative of resistance with respect to magnetic field ($d^2R/dB^2$), however, two oscillations of different periods are observed (Supplementary Figure S4b), corresponding to the SdH oscillations in the gated and ungated regions [4]. The periods of the SdH oscillations ($\Delta(1/B)$) were obtained from the FFT spectrum of $d^2R/dB^2$. As shown in Supplementary Figure S4c, the two peak frequencies correspond to the periods of 0.100 $T^{-1}$ and 0.074 $T^{-1}$. Using the relationship: $\Delta(1/B) = 2e/n_sh$, where $e$ is the elementary charge and $h$ is Planck's constant, the electron concentrations, $n_s$ are calculated to be $4.9{\times}10^{15}$ $m^{-2}$ and 6.5 $\times 10^{15}$ $m^{-2}$, respectively.

By measuring the resistance as a function of magnetic field at different $V_g$, the $V_g$ dependence of $n_s$ was obtained. For $V_g$ = 0 V, only one SdH oscillation period ($\Delta(1/B)$ = 0.074 $T^{-1}$) was observed, with a

corresponding $n_s$ of 6.5 $\times10^{15}$ m$^{-2}$, indicating that the gated and ungated regions have the same $n_s$ in this case. As $V_g$ is swept below –1.4 V, two peaks emerge in the FFT spectrum of $d^2R/dB^2$. As $V_g$ decreases further, the peak at the higher frequency remains constant (as it arises from the ungated region) while the lower frequency peak gradually decreases in frequency (since it arises from the gated region). As shown in Supplementary Figure S4d, $n_s$ in the ungated region remains approximately constant at a value of ~$6.5\times10^{15}$ m$^{-2}$, while the $n_s$ in the gated region gradually decreases from $6.5\times10^{15}$ m$^{-2}$ for $V_g$ = 0 V, to $3.6\times10^{15}$ m$^{-2}$ for $V_g$ = –2.5 V.

**Supplementary Note 5: Dependence of gate-modulation signals on the amplitude of $V_{mod}$ and temperature.**

To select the optimum value of $V_{mod}$ for measurements, gate-modulation signals with different amplitudes of $V_{mod}$ were compared. Measurements were taken at a constant temperature of 2 K using the same experimental arrangement shown in Figure 1a of the main article. $V_{DC}$ was set to be +5 V, and the average powers of both pump and probe beam were 2 mW. When $V_g$ is around –2 V, the amplitude of the plasmonic oscillation is the strongest (Figure 4a of the main article), and so this point was selected for the optimisation.

During data processing, the measured gate-modulation signals for different $V_{mod}$ were divided by the corresponding amplitude $V_{mod}$ for normalization and filtered by a low-pass filter with a cut-off frequency of 0.6 THz to remove high-frequency noise. The gate-modulation signals for different $V_{mod}$ (10, 25, 50 and 100 mV rms) are shown in Supplementary Figure S5a. When the amplitude of $V_{mod}$ was increased from 10 to 100 mV rms, the two periodic oscillations are still apparent, but the oscillations are observed to decay faster for $V_{mod}$ = 100 mV rms than at lower values of $V_{mod}$. In the corresponding frequency spectra, the first and second gated plasmon modes are all discernible and are almost constant (Supplementary Figure S5b). However, the plasmonic resonances for $V_{mod}$ = 100 mV rms are not as sharp as the signals for smaller $V_{mod}$, in accordance with the larger decay rate of oscillations in the time domain.

The difference in decay rates for different $V_{mod}$ can be explained by a change in quality ($Q$) factor of the gated plasmonic cavity. In the gated plasmonic cavity under application of a DC bias, the $Q$ factor $Q = f_r/\Delta f_0$, where $f_r$ is the resonance frequency and $\Delta f_0$ is the half power bandwidth. Here, $\Delta f_0$ is determined by the losses in the Fabry-Pérot cavity, principally ohmic losses in the cavity and coupling

losses at the cavity boundaries. When $V_{mod}$ is applied in addition to $V_g$, the plasmonic resonance frequencies are tuned dynamically around the centre frequency $f_r$, and the tuning range is denoted by $\Delta f_{mod}$. The resonance bandwidth of the gated plasmonic cavity is then widened by $\Delta f_{mod}$. In this case, the $Q$ factor of the gated plasmonic cavity, which is being modulated by $V_{mod}$, can be given by $Q = f_r/(\Delta f_0 + \Delta f_{mod})$. Based on Equation (1) of the main article, we obtain

$$\mathrm{df} \propto \frac{f}{n_s} \cdot dn_s \qquad \text{(s1)}$$

where $f$ is the gated plasmon frequency. For constant $V_g$, the change of $n_s$ ($\Delta n_s$) increases monotonically with the amplitude of $V_{mod}$. Therefore, the increase of amplitude of $V_{mod}$ results in an increase of $\Delta f_{mod}$, and the Q factor correspondingly decreases. As a result, plasmonic oscillations for $V_{mod}$ = 100 mV rms exhibit the largest decay rate observed in the time-domain.

Based on the above analysis, a trade-off between the SNR and accuracy of the plasmon resonance frequency measurement is required in order to select $V_{mod}$ in gate-modulation measurements. For example, in our measurements, the amplitude of $V_{mod}$ was set to be between 25 and 100 mV rms. When the plasmonic oscillation was strong, *e.g.* when $V_g$ = –2 V and the magnetic field is 0 T, we chose $V_{mod}$ = 25 mV rms in order to obtain precise plasmon resonance frequencies. As $V_g$ approaches 0 V, the plasmonic oscillation becomes relatively weak, so a large $V_{mod}$ is needed to improve SNR, and $V_{mod}$ = 100 mV rms was usually used.

To study the temperature dependence, the gate-modulation signals were measured as a function of temperature. As shown in Supplementary Figure S5c, the gate-modulation signals for $V_g$ = –2 V are very similar at the three measured temperatures of 2 K, 4.5 K, and 10 K, implying that the excitation and propagation of 2D plasmons in the 2DES mesa does not change significantly in the temperature range 2–10 K. In the corresponding frequency spectra (Supplementary Figure S5d), the plasmonic resonance frequencies of the first and second modes in the gated regions do not show obvious change as the temperature increases from 2 K to 10 K, indicating that $n_s$ does not change significantly in this temperature range.

**Supplementary Note 6: Signal-to-noise ratio (SNR) of the gate-modulation technique**

As discussed in the main article, the signal measured using the gate-modulation technique can be approximated to $\mathrm{d}I(t)/\mathrm{d}V_g$. Here, we compare the SNR of $\mathrm{d}I(t)/\mathrm{d}V_g$ for $V_g$ = –2.5 V using using both the difference pulse, and the gate-modulation technique. For both methods, measurements were

performed in a liquid-helium continuous flow cryostat at 4 K, with the same setup for coplanar mode excitation. For the difference pulse method, the measured transmitted coplanar mode pulses for $V_g$ = –2.4 V and –2.6 V are shown in Supplementary Figure S3b. d$I(t)$/d$V_g$ for $V_g$ = –2.5 V was obtained by the central difference: ($I(t)(V_g$ = –2.4 V) – $I(t)(V_g$ = –2.6 V))/((–2.4 V) – (–2.6 V)). For the gate-modulation technique, an AC voltage ($V_{mod}$) of amplitude 100 mV rms was superimposed onto $V_g$, whilst the optical chopper used to modulate the probe beam in the previous measurements was removed. In this case, the modulated signal for $V_g$ = –2.5 V was measured at S2 using a lock-in amplifier with reference signal supplied from the AC voltage source.

Signals measured using the difference pulse method and gate-modulation technique are normalized and plotted in Supplementary Figure S6. The shape of the gate-modulation signal resembles closely that of the difference pulse signal, but a significant improvement in SNR is observed. Considering that the delay time for THz in CPW is 9.8 ps, the real signal measured at times earlier than 9.8 ps should be zero (Figure 2a of the main article). We can therefore use the time-domain signal fluctuation observed in the time window from 0 to 9.8 ps to study the noise levels of the two methods. The fluctuations in signal observed when using the difference pulse method are in excess of 10%. When using the gate-modulation technique, however, these fluctuations are reduced to only 0.2%, so demonstrating a 50 times improvement in SNR

**Supplementary Note 7: Theoretical model of gate-modulation technique**

Here, we present a model to discuss our gate-modulation technique. The input pulse is represented in terms of its spectral density by $I_0(\omega)$. The transmitted pulse $I(\omega)$ is denoted by $I(\omega) = I_0(\omega)T(\omega,V_g)$, where $T(\omega,V_g)$ is the overall transmission coefficient, which depends upon the gate voltage. If there is only one $V_g$ dependent resonant excitation present in the cavity, $T(\omega,V_g)$ can be represented by:

$$T(\omega,V_g) = B(\omega,V_g)\frac{1}{\omega-\omega_0(V_g)+i\gamma(V_g)}\,, \qquad \text{(s2)}$$

where $B(\omega,V_\mathrm{g})$ is used to describe the broadband non-resonant effects which includes the contribution of leads, junctions, *etc,* $\omega_0(V_\mathrm{g})$ is the resonant frequency, and γ is decay rate. Therefore, the transmitted pulse can be represented by the following formula:

$$I(\omega,V_g) = I_0(\omega)B(\omega,V_g)\frac{1}{\omega-\omega_0(V_g)+i\gamma(V_g)}. \qquad \text{(s3)}$$

For gate-modulation technique, the measured signal is equivalent to the derivative of transmitted signal if the amplitude of $V_{mod}$ is sufficiently small,

$$\tilde{I}_\omega = \frac{dI(\omega,V_g)}{dV_g}\tilde{V}_g = \tilde{V}_g I_0(\omega)\frac{\partial}{\partial V_g}\left(B(\omega,V_g)\frac{1}{\omega-\omega_0(V_g)+i\gamma(V_g)}\right). \quad \text{(s4)}$$

Thus, the extracted signal can be equivalent to a sum of two terms

$$\tilde{I}_\omega = \frac{I_0(\omega)\tilde{V}_g B(\omega,V_g)}{\left(\omega-\omega_0(V_g)+i\gamma(V_g)\right)^2}\left[\frac{d\omega_0}{dV_g} - i\frac{d\gamma}{dV_g}\right] + \frac{dB(\omega,V_g)}{dV_g}\frac{I_0(\omega)\tilde{V}_g}{\omega-\omega_0(V_g)+i\gamma(V_g)}. \quad \text{(s5)}$$

If the resonance is very sharp, $\omega_0(V_g) > \gamma(V_g)$, there will be a strong peak in the spectrum around $\omega = \omega_0(V_g)$ and the first terms plays a dominant role. In that case, the measured gate modulation signals around $\omega = \omega_0(V_g)$ can be approximated as

$$\tilde{I}_\omega = \frac{I_0(\omega)\tilde{V}_g B(\omega,V_g)}{\left(\omega-\omega_0(V_g)+i\gamma(V_g)\right)^2}\left[\omega_0' - i\gamma'\right], \quad \text{(s6)}$$

where $\omega'_0 = d\omega_0/dV_g$, $\gamma' = d\gamma/dV_g$. Correspondingly, the absolute value is

$$|\tilde{I}_\omega| = |I_0(\omega)||\tilde{V}_g||B(\omega,V_g)|\frac{\sqrt{\omega_0'^2+\gamma'^2}}{(\omega-\omega_0(V_g))^2+\gamma(V_g)^2}. \quad \text{(s7)}$$

The maximum appears at $\omega = \omega_0(V_g)$ and the linewidth is determined by $\gamma$, the corresponding height is

$$|\tilde{I}_\omega^{max}| = |I_0(\omega)||\tilde{V}_g||B(\omega,V_g)|\frac{\sqrt{\omega_0'^2+\gamma_0'^2}}{\gamma(V_g)^2}. \quad \text{(s8)}$$

Based on the above model, the gate-modulation method provides an effective way to study the low loss resonant excitation in the 2DES cavity. For the plasmon mode in the gated region, $\gamma$ is low unless the gated region is strongly depleted. Therefore, we could observe the periodic oscillations with the same frequency as plasmon resonance mode from the gate-modulation signals.

The model can also be used to explain the extraction of ungated plasmon resonances. For an ungated plasmon resonance, the first term in Equation (s5) is 0 as $\omega$ and $\gamma$ do not change with $V_g$. Thus,

$$\tilde{I}_\omega = \frac{dB(\omega,V_g)}{dV_g}\frac{I_0(\omega)\tilde{V}_g}{\omega-\omega_0+i\gamma}. \quad \text{(s9)}$$

The measured signal is determined by $B(\omega,V_g)$, which corresponds to the transmission coefficient of the broadband non-resonant signal. Based on the measured results shown in Figure 2b of the main article, the derivative $B(\omega,V_g)$ with respect to $V_g$ cannot be overlooked when $V_g$ is below –2 V. In that case, a peak appears at the ungated plasmon resonance frequency in the spectrum of gate-modulation signals and its frequency does not change with $V_g$.

## Supplementary Figures

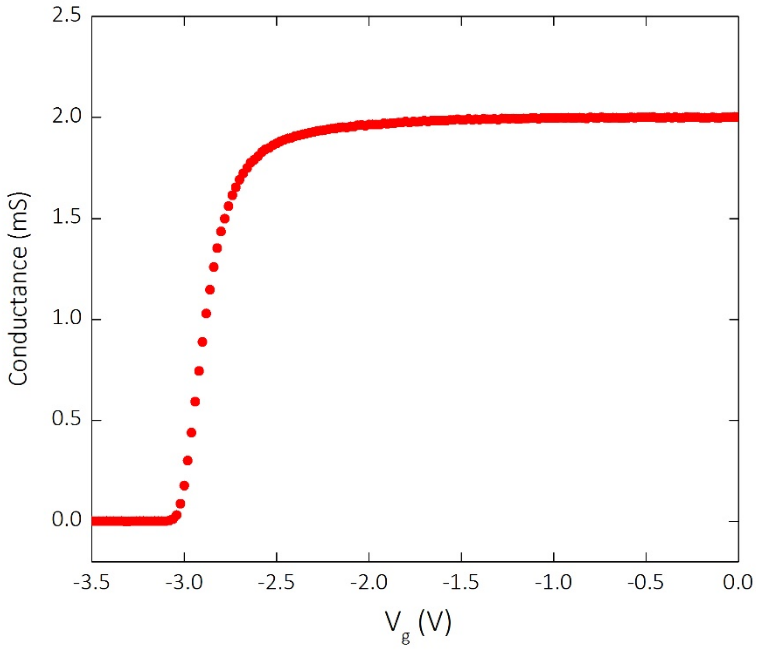


**Supplementary Figure S1. Measured conductance of the 2DES mesa as a function of gate voltage $V_g$ at 2 K.** The resistance of the leads and centre conductor of the coplanar waveguide (CPW) are not subtracted.

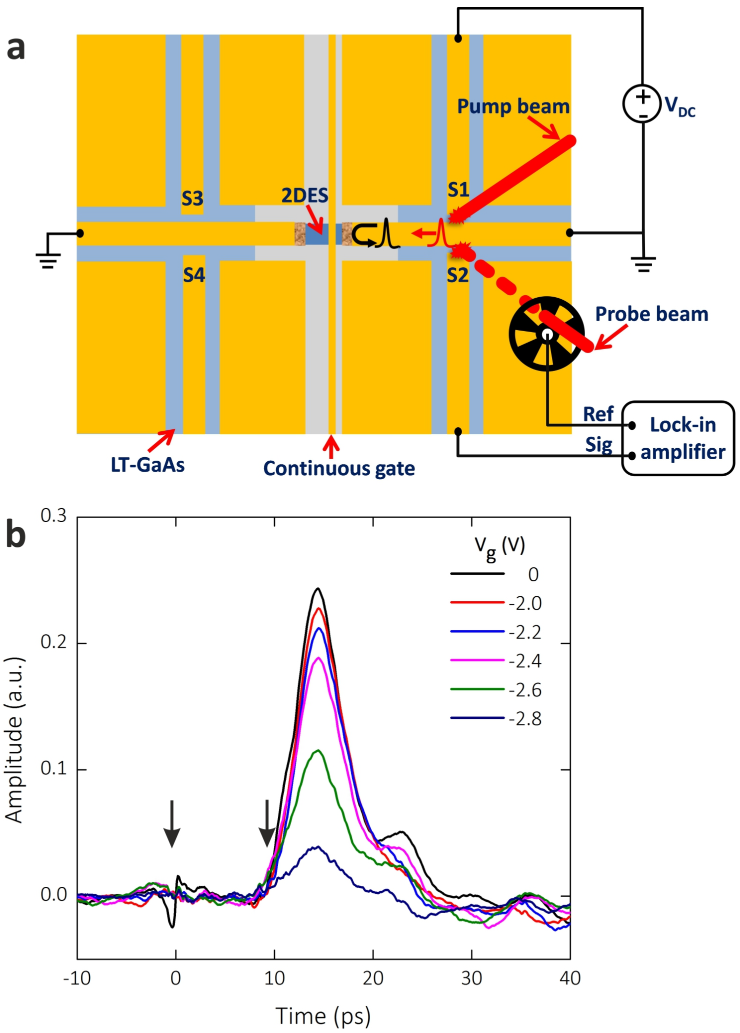


**Supplementary Figure S2. Input and reflected THz pulse measurement at 4 K.** (a) The experimental arrangement used to measure the input and reflected THz pulses. A THz pulse is generated at switch S1, which is illuminated by a near-infrared (NIR) pump laser beam and biased by a positive DC voltage ($V_{DC}$). Switch S2 is illuminated by an optically chopped and time-delayed probe laser beam, allowing detection of the pulse generated at S1 and the pulse reflected from the 2DES mesa. The current induced in S2 by the THz frequency electric field is recorded by a lock-in amplifier, referenced to the optical chopper frequency. (b) The change in the reflected signal relative to that found at $V_g = -3$ V for different $V_g$ at 4 K, and obtained by subtracting the signal at $V_g = -3$ V in Figure 2a of the main article from each signal recorded at different $V_g$. The peak positions of input pulses (0 ps) and the reflected pulses from ohmic contact (9.8 ps) in Figure 2a of the main article are indicated using black arrows.

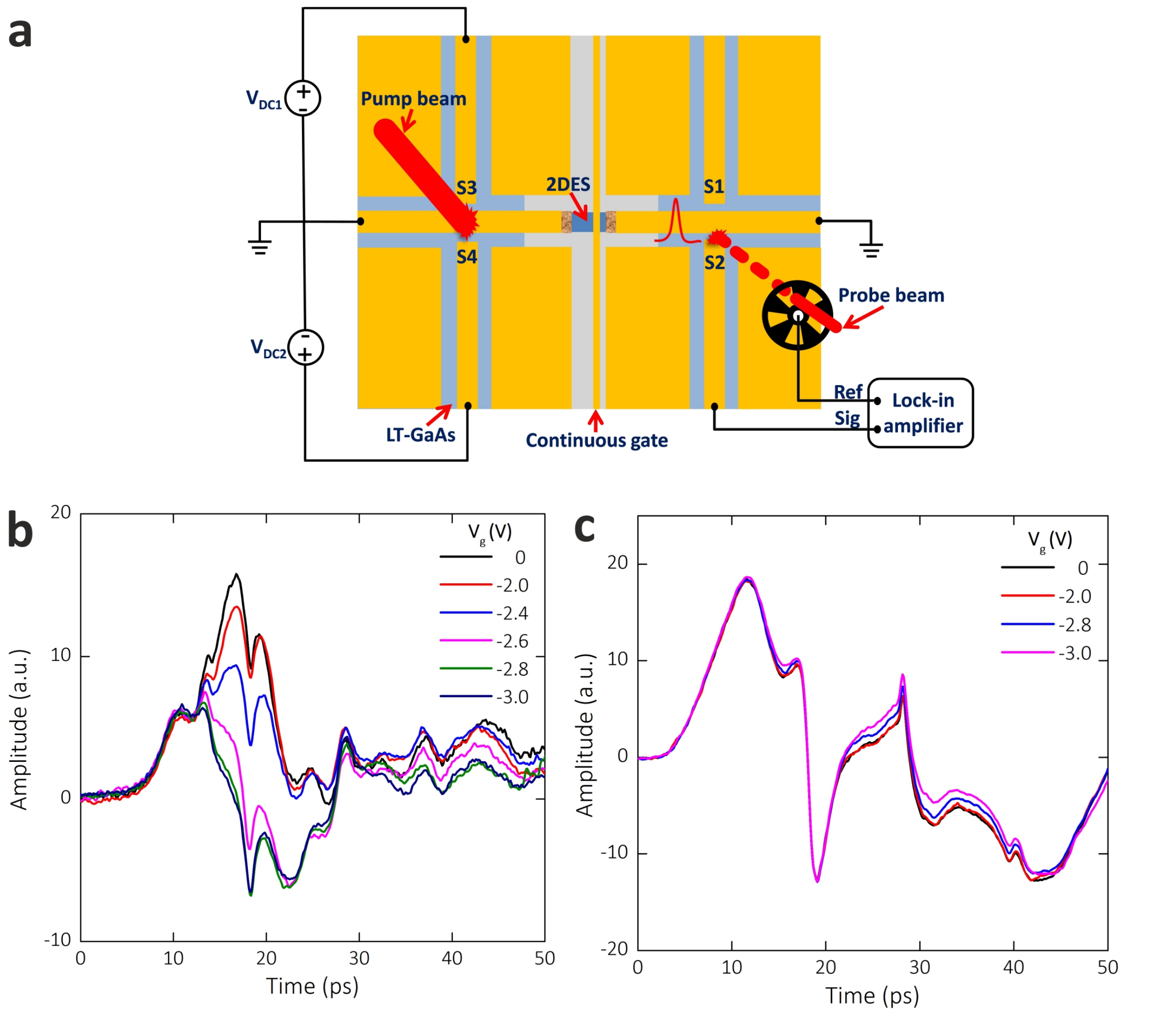


**Supplementary Figure S3. Dependence of the transmitted coplanar mode and slotline mode signals in the CPW on $V_g$.** (a) The experimental apparatus used for selective mode generation and transmitted signal measurements. Adjacent PC switches located on one side of the mesa (*e.g.* S3 and S4) are illuminated by a defocused pump laser beam, and are biased with $V_{DC1}$ and $V_{DC2}$, respectively, to select a particular mode. To excite a coplanar mode, $V_{DC1} = V_{DC2}$, while to excite the slotline mode, $V_{DC1} = -V_{DC2}$. Transmitted pulses are detected by illuminating a third PC switch, located on the opposite side of 2DES mesa (*e.g.* S2), using a time-delayed and optically chopped probe beam. (b) The transmitted coplanar mode signals ($V_{DC1} = V_{DC2} = +20$ V) for different $V_g$ at 4 K. (c) The transmitted slotline mode signals ($V_{DC1} = -V_{DC2} = +20$ V) for different $V_g$ at 4 K.

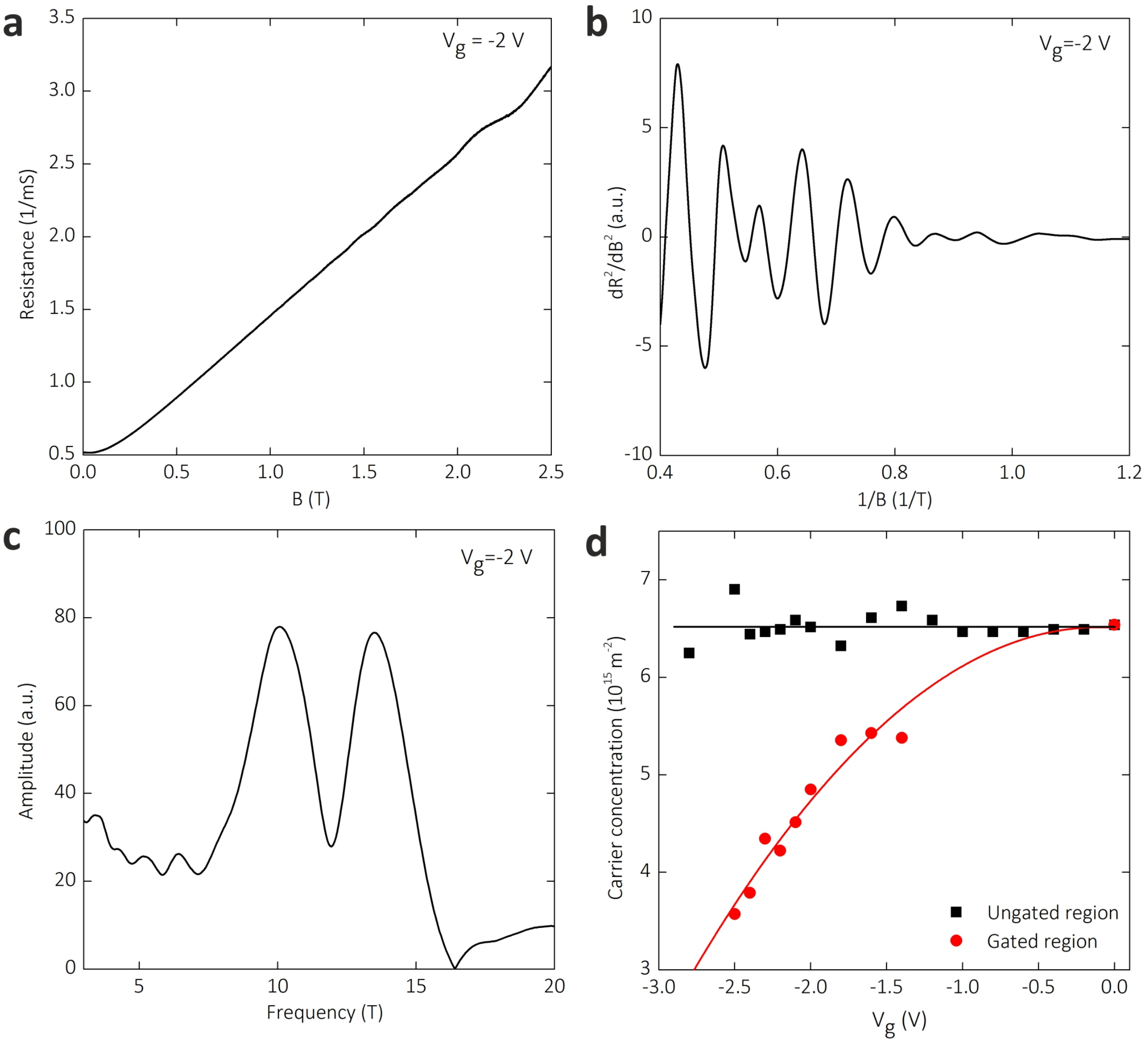


**Supplementary Figure S4. Two-terminal magnetotransport measurements and determination of the electron concentration (*$n_s$*) in the 2DES mesa.** (a) The two-terminal resistance of the 2DES mesa as a function of magnetic field (*B*), in which the resistance of the leads and the centre conductor of the CPW are not subtracted. (b) The second derivative of *R* with respect to *B* ($d^2R/dB^2$) as a function of 1/*B*. (c) The fast Fourier transform (FFT) spectrum of $d^2R/dB^2$ shown in (b). (d) Calculated $n_s$ in the ungated (black squares) and gated (red circles) regions as a function of $V_g$. The solid lines are to guide the eye and indicate that $n_s$ in the ungated region (black) is constant, while $n_s$ in the gated region (red) decreases, when $V_g$ decreases from 0 V to –3 V.

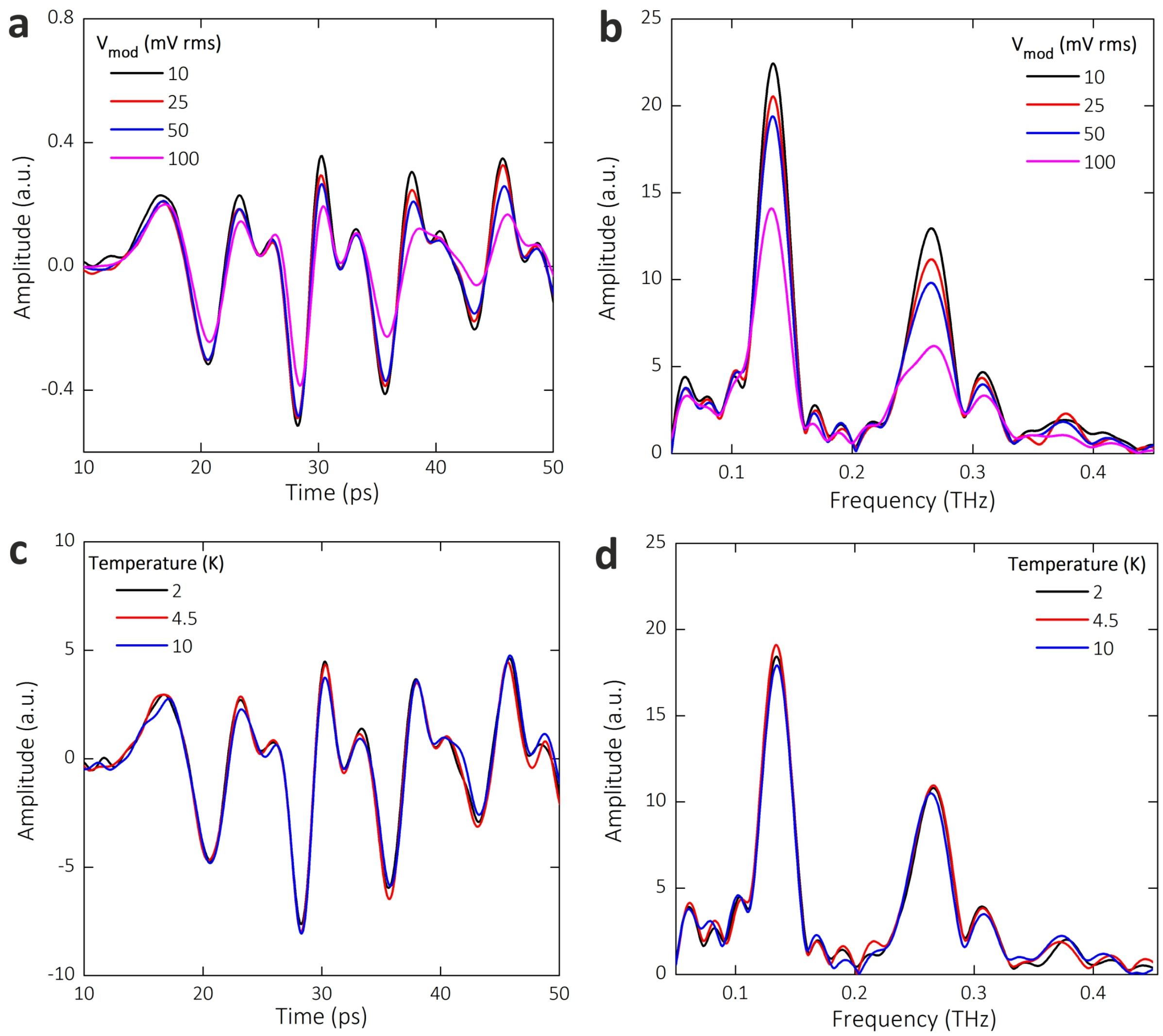


**Supplementary Figure S5. Dependence of the gate-modulation signals on $V_{mod}$ and temperature.** (a) The measured gate-modulation signals for $V_g$ = –2 V at 2 K with different amplitudes of $V_{mod}$, ranging from 10 mV rms to 100 mV rms. Each signal is divided by the corresponding amplitude of $V_{mod}$. (b) The corresponding FFT spectra of the signals in (a). (c) The measured gate-modulation signals for $V_g$ = –2 V at different temperatures. (d) The corresponding FFT spectra of the signals in (c).

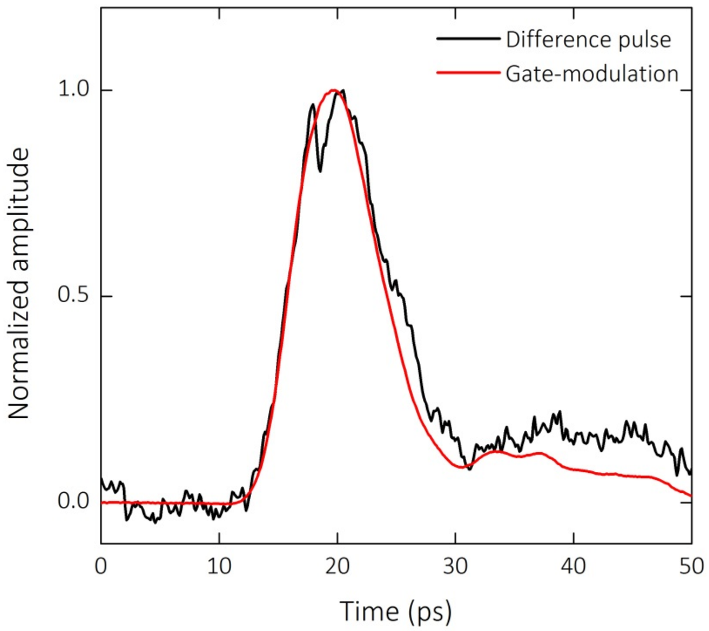


**Supplementary Figure S6. Comparison of the derivative of transmitted pulse with respect to $V_g$ ($dI(t)/dV_g$) for $V_g$ = –2.5 V using the difference pulse method and gate-modulation technique.**

## Supplementary References


1. Davies, J. H. *The Physics of Low-dimensional Semiconductors: An Introduction Ch. 9* (Cambridge Univ. Press, Cambridge, 1998).
2. McGowan, R. W., Grischkowsky, D. & Misewich, J. A. Demonstrated low radiative loss of a quadrupole ultrashort electrical pulse propagated on a three strip coplanar transmission line. *Appl. Phys. Lett.* **71**, 2842-2844 (1997).
3. Zamdmer, N., Hu, Q., Verghese, S. & Förster. A. Mode-discriminating photoconductor and coplanar waveguide circuit for picosecond sampling. *Appl. Phys. Lett.* **74**, 1039-1041 (1999).
4. Chang, C. S., Fetterman, H. R. & Viswanathan, C. R. The characterization of high electron mobility transistors using Shubnikov–de Haas oscillations and geometrical magnetoresistance measurements. *J. Appl. Phys.* **66**, 928-936 (1989).
